# Levels of HBV RNA in chronic HBV infected patients during first-line nucleos(t)ide analogues therapy

**DOI:** 10.1186/s13027-022-00473-9

**Published:** 2022-12-07

**Authors:** Bei Jiang, Qinghai Dai, Yamin Liu, Guangxin Yu, Yuqiang Mi

**Affiliations:** 1 Tianjin Second People’s Hospital, Tianjin Medical University, Tianjin, 300192 People’s Republic of China; 2 Tianjin Institute of Hepatology, Tianjin Second People’s Hospital, 7th Sudi South Road, Nankai, Tianjin, 300192 People’s Republic of China; 3 Department of Microbiology and Infectious Disease Center, School of Basic Medical Sciences, Peking University Health Science Center, Beijing, 100191 People’s Republic of China

**Keywords:** HBV RNA, Hepatitis B virus, Nucleos(t)ide analogues, HBsAg, HBV DNA

## Abstract

**Background:**

Serum HBV RNA has been considered a potential biomarker in monitoring the prognosis of chronic hepatitis B (CHB). However, Real-life cohort studies on the profile of HBV RNA in chronic HBV infected patients during first-line nucleos(t)ide analogues (NAs) are lacking. We aimed to investigate HBV RNA dynamic pattern and clinical value chronic HBV infected patients under NA therapy.

**Methods:**

HBV RNA and clinical assessments were measured in 82 treatment-naïve chronic HBV infected patients. These enrolled patients were categorized into HBeAg-positive chronic HBV infected (n = 53) and HBeAg-negative chronic HBV infected (n = 29). Of these, there were 59, 46, and 30 chronic HBV infected patients completed the follow-up clinical assessments at 12, 24, and 48 weeks of NAs therapy, respectively.

**Results:**

In treatment-naïve patients, there was a positive correlation between HBV RNA and HBV DNA, HBsAg (r = 0.602 and 0.502. *P* < 0.05). The median level of HBV DNA was higher than HBV RNA by 1.64 log_10_ copies/mL_._ The mean level of serum HBV RNA was 4.62 (IQR: 3.05–5.82) log_10_ copies/mL at baseline, and the median level of HBV RNA was 2.88 (IQR: 0–4.67), 2.71 (IQR: 0–4.22), and 2.96 (IQR: 0–4.32) log_10_ copies/mL at week 12, 24, and 48, respectively. HBV RNA showed a positive linear correlation with HBV DNA at 12, 24, and 48 weeks of NA treatment (r = 0.640, 0.715, and 0.656 respectively, *P* < 0.05). In patients who were treated 48 weeks NAs, 67% had quantifiable HBV RNA while only 37% had quantifiable HBV DNA.

**Conclusion:**

HBV RNA has signature profiles in different stages of chronic HBV infected patients receiving first-line NAs. During antiviral treatment, HBV RNA can still monitor the virus activity in patients whose serum HBV DNA cannot be detected.

**Supplementary Information:**

The online version contains supplementary material available at 10.1186/s13027-022-00473-9.

## Introduction

Hepatitis B virus (HBV) infection is still a serious global public health problem [1]. Nucleos(t)ide analogues (NAs) are being used widely to treat chronic hepatitis B (CHB) patients [2]. NAs as chain terminators to inhibit reverse transcription of pregenomic RNA (pgRNA) into HBV DNA, but do not immediately reduce the covalently closed circular DNA (cccDNA) pool [3]. Therefore, HBV infection cannot be eliminated due to the persistence of cccDNA in the nuclei of infected hepatocytes. Although negative serum HBsAg is considered the standard of functional therapy for CHB, the number of patients reaching the ideal treatment endpoint is far from satisfactory [4]. Liver biopsies to quantitate intrahepatic cccDNA are invasive and cannot be repeatedly performed as a routine diagnosis [5]. Serum HBV DNA quantification has been used to monitor viral activities. However, with the availability of first-line NAs, serum HBV DNA rapidly becomes undetectable. Therefore, it is necessary to explore noninvasive surrogate markers to reflect the intrahepatic cccDNA activity and assess disease phase. Recently, growing studies suggest that HBV RNA may act as a novel serum biomarker for HBV infection, treatment and prognosis [6–8].

According to the HBV life cycle profile, Serum HBV RNA is transcribed from cccDNA in the infected hepatocytes. It has been proved that pgRNA is encapsulated in HBV core particles, thereafter reverse transcribed to the HBV DNA mostly and the mature particles are enveloped and released to the serum. Meanwhile, a certain level of pgRNA which was non- or partially reverse transcribed could also be enveloped and released as HBV RNA virion-like particles[9]. Some studies have inferred that serum HBV RNA can be a useful marker for monitoring the efficacy of antiviral therapy [10, 11], because the level of serum HBV RNA in patients receiving NAs therapy can reflect the presence of the cccDNA and its transcriptional activity in hepatocytes. Moreover, the first-line anti-viral agent such as NAs can reduce the serum HBV DNA down to undetectable level but does not immediately influence the quantification of HBsAg, HBcrAg (hepatitis B core -related antigen), and HBV RNA [11]. In this respect, especially in virally suppressed patients with undectable HBV DNA under NAs therapy, serum HBV RNA has been considered a new biomarker to monitor antiviral efficacy [12]. In fact, HBV RNA levels vary with the phase of infection clinically. Thus far, it is needed to delineate molecular details of serum HBV RNA during different stages of chronic HBV infected natural history, from baseline to different treatment time points receiving NAs therapy.

In this study, we evaluated the parameters and distribution patterns of serum HBV RNA, HBV DNA, and HBsAg in a prospective cohort study of chronic HBV infected patients treated with NAs at baseline and at week 12, 24, and 48, and analyzed the correlation between these serological indicators. These results enable us to distinguished better the clinical characteristics of HBV RNA based on different chronic HBV infected stages under NAs therapy. The promising findings showed preliminary clinical significance and clinical potentials of HBV RNA.

## Methods

### Enrolled patients

A total of 82 treatment-naïve patients with chronic HBV infection were enrolled in a prospective cohort study from Tianjin Second People’s Hospital. These patients began first-line NAs (entecavir or tenofovir) therapy and were followed up for 12, 24, and 48 weeks between December 2018 and November 2020. The diagnosis of clinical specimens is based on the 2015 Guidelines to prevent and treat chronic hepatitis B [13]. The inclusion criteria as follows: (a) patients with detectable HBsAg and/or HBV DNA for more than six months before treatment; (b) treatment-naïve patients who had detectable HBsAg and/or HBV DNA for more than six months before treatment; (c) patients willing to provide informed consent. The exclusion criteria for the patients were as follows: (a) patients who had severe liver-related complications (including decompensated liver cirrhosis, hepatocellular carcinoma, and liver transplantation); (b) patients with human immunodeficiency virus (HIV) or hepatitis C virus (HCV) co-infection. The studies described above are in line with the Helsinki Declaration's ethical principles and were approved by the Ethics Committee of Tianjin Second People’s Hospital (Approved No. 2018 [15]). Each patient enrolled signed an informed consent form.

### Laboratory measurements

A Hitachi 7180 automatic biochemical analyzer (Japan) performed routine biochemical tests during therapy at the follow-up point. Serum HBsAg levels, antibody to HBsAg (HBsAb), HBeAg, and antibody to HBeAg (HBeAb) were determined by an Architect I2000SR electrochemistry luminescence immunity analyzer (U.S.A). HBsAg and HBsAb levels were expressed in IU/mL and mIU/mL and had dynamic detection ranges which are 0–124,950 IU/mL and 0–1000 mIU/mL, respectively. HBeAg and HBeAb were determined semi-quantitatively and expressed as the signal-to-cut-off ratio (S/CO). Liver stiffness measurement (LSM) was assessed by FibroScan-502 (Echosens, Paris, France), equipped with an M-type probe at 3.5 MHz. The result for LSM was presented in kPa.

### Measurement of serum HBV DNA

The COBAS TaqMan assay (Roche Diagnostics) was amplified to quantify the serum HBV DNA loads with a detection range of 20–1.7 × 10^8^ IU/mL. The negative HBV DNA was defined as less than 20 IU/mL.

### Measurement of serum HBV RNA

Serum HBV RNA was extracted and quantified with HBV RNA extraction and detection kit (Hotgen, Beijing, China). Briefly, HBV RNA was treated with DNase I after extraction process to remove the interference of HBV DNA as previous studies suggested [8]. The levels of HBV RNA were detected by RT-qPCR in Applied Biosystems 7500 Real-Time PCR Systems (Applied Biosystems, Mannheim, USA) with a TaqMan probe method following the manufacturer's protocol. The standard curve for HBV RNA detection was drawn and calibrated with tenfold diluted pseudovirus particles contained HBV RNA standard plasmids which were also provided by HBV RNA extraction and detection kit. The minimum detection limit was 100 copies/mL. The negative HBV RNA was defined as less than 100 copies/mL.

### Statistical analysis

Statistical analyses were performed with R (version 4.0.3, http://www.r-project.org). Continuous variables were expressed in median [interquartile range (IQR)] and were compared using the Mann–Whitney U test. Categorical variables were compared using chi-squared or Fisher’s exact tests. The correlations between two continuous variables were analyzed using the Spearman's rank test. A *P* < 0.05 was considered significant.

## Results

### Characterization of the enrolled patients

**Fig. 1:**
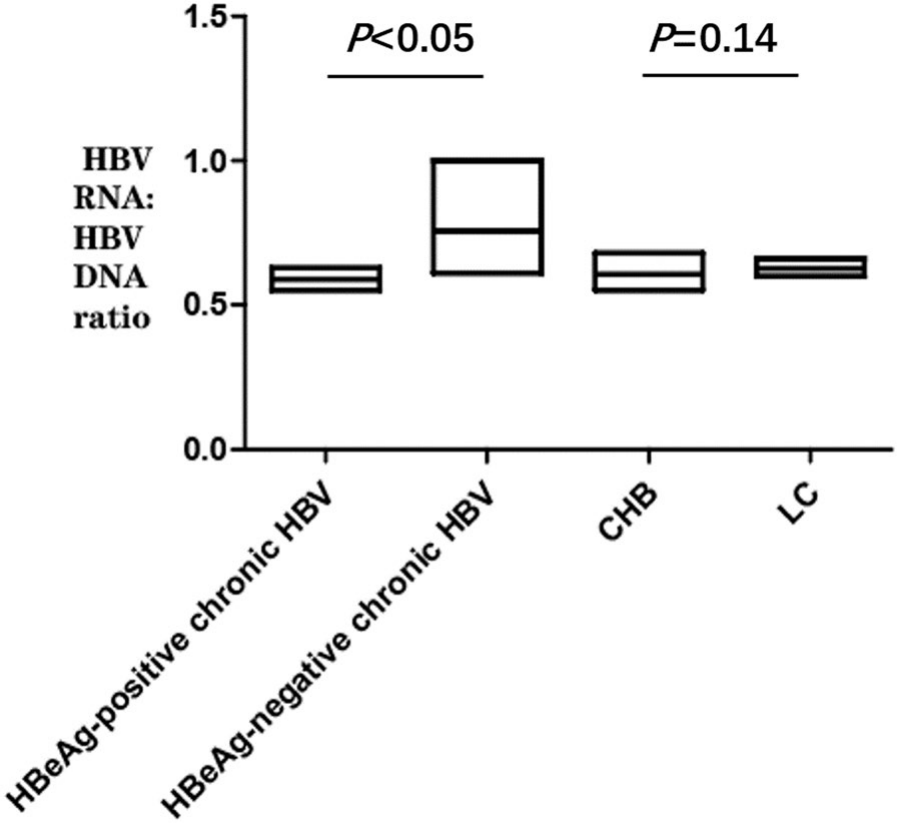
Serum HBV RNA to HBV DNA ration in chronic HBV infection

WE enrolled a total of 82 chronic HBV infected patients who were treatment- naïve. These were categorized into HBeAg-positive chronic HBV infection (n = 53) and HBeAg-negative chronic HBV infection (n = 29). In addition, according to the clinical diagnosis, these patients were also divided into CHB group (n = 62) and hepatitis B cirrhosis (LC) group (n = 20). The detailed baseline summary characteristics of the included patients are listed in Table 1. The median level of HBV DNA was exhibited to be higher than HBV RNA by 1–2 log_10_ in both HBeAg-positive and HBeAg-negative phases. The ratio of serum HBV RNA to HBV DNA was calculated to assess the reverse transcriptional efficiency of pgRNA [14]. As shown in Fig. 1, The median ratio of HBV RNA to HBV DNA was 0.59 (IQR0.55–0.63), 0.66 (IQR0.61–1.00), 0.59 (IQR0.55–0.68) and 0.62 (IQR0.60–0.66) for HBeAg-positive chronic HBV infection, HBeAg-negative chronic HBV infection, CHB and LC. The RNA to DNA ratio was higher in patients who were HBeAg-negative than HBeAg-positive chronic HBV infection (0.66 vs. 0.59; *P* < 0.05). The ratio was not significantly different for CHB and LC subgroup ((0.59 vs. 0.62; *P* = 0.14). To investigate the utility of HBV RNA in the clinic, we performed an observational, non-interventive, real clinical cohort study. Among the 82 patients who received first-line oral NAs, 59, 46, and 30 patients were followed up for 12, 24, and 48 weeks receiving first-line NAs, respectively. After 48 weeks of follow-up, five patients developed HBeAg seroconversion. No patient had HBsAg seroconversion.

**Table1:** Baseline characteristics of treatment-naïve patients

Parameters	Chronic HBV infection (Baseline)						
	Total (n = 82)	HBeAg positive (n = 53)	HBeAg negative (n = 29)	*P* value	CHB (n = 62)	LC (n = 20)	*P* value
Male (n, %)	55, 67.1%	34, 64.2%	21, 72.4%	0.606	40, 64.5%	15, 75.0%	0.552
Median age (y,range)	43.5(32.2–53.7)	42.0(32.0–54.0)	46.0(36.0–53.0)	0.223	38.5(31.2–49.7)	55.0(45.5–62.2)	< 0.05*
Median albumin (g/L,IQR;range)	44.4(39.9–46.5)	43.4(38.7–45.9)	46.2(43.3–48.7)	< 0.05*	45.2(42.7–46.7)	37.4(32.0–45.1)	< 0.05*
Median bilirubin (μmol/L, IQR;range)	16.0(12.7–24.3)	16.4(12.9–27.2)	15.4(11.4–18.4)	0.322	16.0(12.2–21.0)	16.1(14.3–30.2)	0.359
Median ALT (U/L,IQR;range)	66.5(34.5–121.5)	71.0(45.0–141)	42.0(24.0–94.0)	0.082	72.0(40.5–159.8)	66.5(34.5–121.5)	0.024*
Median AST(U/L, IQR;range) Detectable (n, %) HBV RNA HBV DNA	54.5(35.2–89.5) 71(87%) 81(99%)	63.0(43.0–92.0) 52(98%) 53(100%)	45.0(27.0–63.0) 19(66%) 28(97%)	0.032*	54.0(36.0–94.3) 52(84%) 61(98%)	60.0(35.0–70.5) 19(95%) 20(100%)	0.574
Median HBV RNA (log10 copies/mL)	4.62(3.05–5.82)	5.57(4.07–6.43)	2.71(0.00–4.01)	< 0.05*	4.71(2.84–6.12)	3.88(3.22–4.96)	0.336
Median HBV DNA (log10 IU/mL)	6.26(5.20–7.21)	7.02(5.54–8.22)	4.88(3.19–6.10)	< 0.05*	6.36(5.25–7.67)	6.03(4.95–7.03)	0.489
Median qHBsAg (log10 mIU/mL)	3,62(3.35–4.03)	3.84(3.40–4.22)	3.48(3.08–3.67)	< 0.05*	3.67(3.36–4.18)	3.53(3.34–3.72)	0.063
Median LSM (kPa)	11.80(6.85–17.88)	11.90(8.00–21.30)	10.20(6.70–14.50)	0.182	10.00(6.38–13.85)	21.90(14.45–36.03)	< 0.05*

^*^P < 0.05 is considered significant difference between two groups

### Correlation between HBV serological markers in treatment-naïve patients

We first analyzed the correlation coefficients between HBV serological markers in treatment-naïve patients. The summary of correlation coefficients between HBV serological markers in treatment-naïve patients are shown in Table 2. Among the total 82 patients at baseline, the serum levels of HBV RNA were positively associated with HBV DNA (r = 0.602, *P* < 0.05), HBsAg (r = 0.502, *P* < 0.05). In addition, we found a positive correlation between HBV DNA and HBsAg (r = 0.496, *P* < 0.05). Furthermore, we also analyzed the correlations of HBV serological markers in HBeAg-positive and HBeAg-negative chronic HBV infection subgroups. a moderate positive correlation of HBV RNA level and HBV DNA was also observed in either HBeAg-positive patients (r = 0.445, *P* < 0.05) or HBeAg-negative patients (r = 0.478, *P* < 0.05) at baseline. The levels of HBV RNA and HBsAg remained correlated in HBeAg-positive patients (r = 0.413, *P* < 0.05), but in HBeAg-negative patients there was no correlation (r = 0.352, *P* = 0.061). Moreover, there was a linear correlation with HBsAg and HBV DNA in HBeAg-positive patients (r = 0.442, *P* < 0.05) but not in HBeAg-negative patients (r = 0.265, *P* = 0.165). Further subgroup analysis was performed based on clinical diagnosis. In the CHB group, HBV RNA showed a positive linear correlation with HBV DNA and HBsAg (r = 0.598 and 0.515, *P* < 0.05). HBV DNA and HBsAg remained were positively correlated (r = 0.551, *P* < 0.05). Furthermore, in the LC group, HBV RNA only showed a strong linear correlation with HBV DNA (r = 0.700, *P* < 0.05), but not with HBsAg (r = 0.367, *P* = 0.112). There is almost no correlation between HBV DNA and HBsAg (r = 0.278, *P* = 0.235). The correlation plots for HBV serological markers are shown in Additional file 1: Fig. S1, Additional file 2: Fig. S2, Additional file 3: Fig. S3.

**Table 2:** Correlation between HBV serological markers in treatment-naïve patients

	HBV DNA	HBsAg
*All treatment-naïve patients (n = 82)*		
HBV RNA	0.602(*P* < 0.05*)	0.502(*P* < 0.05*)
HBV DNA	–	0.496(*P* < 0.05*)
*HBeAg-Positive (n = 53)*		
HBV RNA	0.445(*P* < 0.05*)	0.413(*P* < 0.05*)
HBV DNA	–	0.442(*P* < 0.05*)
*HBeAg-Negative (n = 29)*		
HBV RNA	0.478(*P* < 0.05*)	0.352(*P* = 0.061)
HBV DNA	–	0.265(*P* = 0.165)
*Chronic hepatitis B (n = 62)*		
HBV RNA	0.598(*P* < 0.05*)	0.515*(P* < 0.05*)
HBV DNA	–	0.551(*P* < 0.05*)
*Liver cirrhosis (n = 20)*		
HBV RNA	0.700(*P* < 0.05*)	0.367(*P* = 0.112)
HBV DNA	–	0.278(*P* = 0.235)

*P* < 0.05 is considered significant difference between two groups

### The proportion of patients with detectable HBV RNA and HBV DNA during 48 weeks of antiviral therapy

**Fig. 2:**
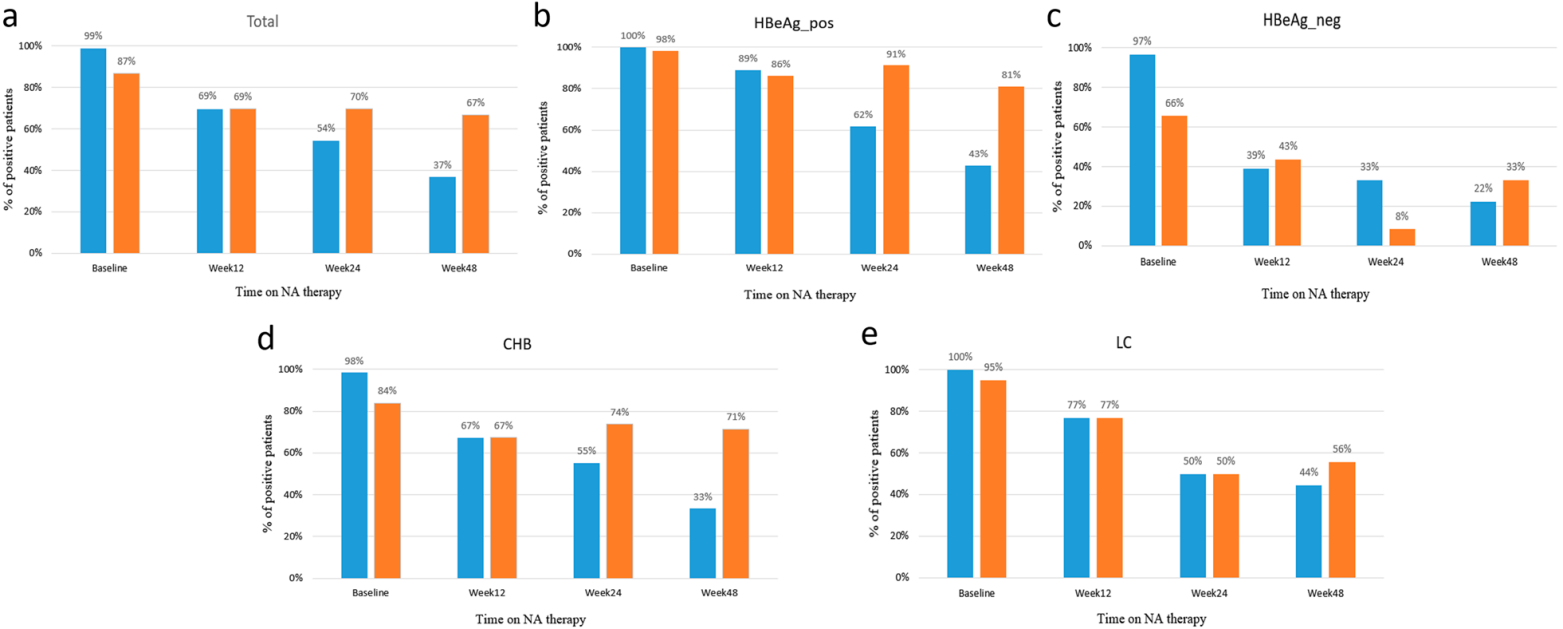
Proportions of patients with detected HBV DNA and HBV RNA during NA therapy in cohort. **a** proportions of total patients. **b** proportions of HBeAg-positive patients. **c** proportions of HBeAg-negative patients. **d** proportions of CHB patients. **e** proportions of LC patients

At therapy baseline, HBV DNA was detected in 99% (n = 81) of enrolled patients, while HBV RNA was only found in 71 (87%) patients. At week 12, 59 patients completed the follow-up. Of those, 41 (69%) patients had detectable HBV DNA, and 41 (69%) patients had detectable HBV RNA. After 24 weeks of therapy, 46 patients completed the follow-up. Among those people, 54% (n = 25) of patients had detectable HBV DNA and 70% (n = 32) had detectable HBV RNA. After 48 weeks of therapy, only 30 patients had completed the follow-up, 37% (n = 11) patients still had detectable HBV DNA, and 67% (n = 20) had RNA (Fig. 2a). Subgroup analysis was performed for HBeAg-positive and HBeAg-negative patients separately (Fig. 2b–c). At baseline, almost all patients (n = 53, 100% for HBeAg-positive; n = 28, 98% for HBeAg-negative) had detectable HBV DNA in serum, while 52 (98%) HBeAg-positive patients and 19 (66%) HBeAg-negative had detectable HBV RNA in serum. At week 12, there were 36 HBeAg-positive patients, and 23 HBeAg-negative patients completed the follow-up. In the HBeAg-positive subgroup, 32 (89%) patients were positive for HBV DNA, and 31(86%) patients were positive for HBV RNA. In the HBeAg-negative subgroup, 9 (39%) patients were positive for HBV DNA, and 10 (43%) patients were positive for HBV RNA. After 24 weeks of NA therapy, there were 34 HBeAg-positive patients, and 12 HBeAg-negative patients completed the follow-up. In the HBeAg-positive subgroup, 21 (62%) patients had detectable HBV DNA, and 31 (91%) patients had detectable HBV RNA. However, in the HBeAg-negative subgroup, HBV DNA was detectable in 4 (33%) patients, and serum HBV RNA was detectable only in one (8%) patient. At week 48, there were 21 HBeAg-positive patients, and 9 HBeAg-negative patients completed the follow-up. In the HBeAg-positive subgroup, 9 (43%) patients were positive for HBV DNA, and 17 (81%) patients were positive for HBV RNA. In the HBeAg-negative subgroup, only 2 (22%) patients were positive for HBV DNA, and 3 (33%) patients were positive for HBV RNA. At the same time, we also analyzed CHB and LC subgroups (Fig. 2d, e). In the CHB subgroup, at baseline, 61 patients (98%) had detectable HBV DNA, and 52 (84%) patients had detectable HBV RNA. At week 12, 46 patients had completed the follow-up. Among them, both of 31(67%) patients had detectable HBV DNA and HBV RNA. At week 24, 38 patients had completed the follow-up. Among them, 21 (55%) and 28 (74%) patients had detectable HBV DNA and HBV RNA, respectively. At week 48, 21 patients completed the follow-up. Among them, 7 (33%) and 15 (71%) patients had detectable HBV DNA and HBV RNA, respectively. In the LC subgroup, at baseline, all patients (n = 20, 100%) had detectable HBV DNA and 19 (95%) patients had detectable HBV RNA. At week 12, 13 patients had completed the follow-up. Among them, both of 10 (77%) patients had detectable HBV DNA and HBV RNA, respectively. At week 24, only eight patients had completed the follow-up. Among them, both of four (50%) patients had detectable HBV DNA and HBV RNA. At week 48, nine patients had completed the follow-up. Among them, 4 (44%) and 5 (56%) patients had detectable HBV DNA and HBV RNA, respectively.

### Dynamic changes of HBV serological markers and biochemical indicators during 48 weeks of NA treatment therapy

**Fig. 3:**
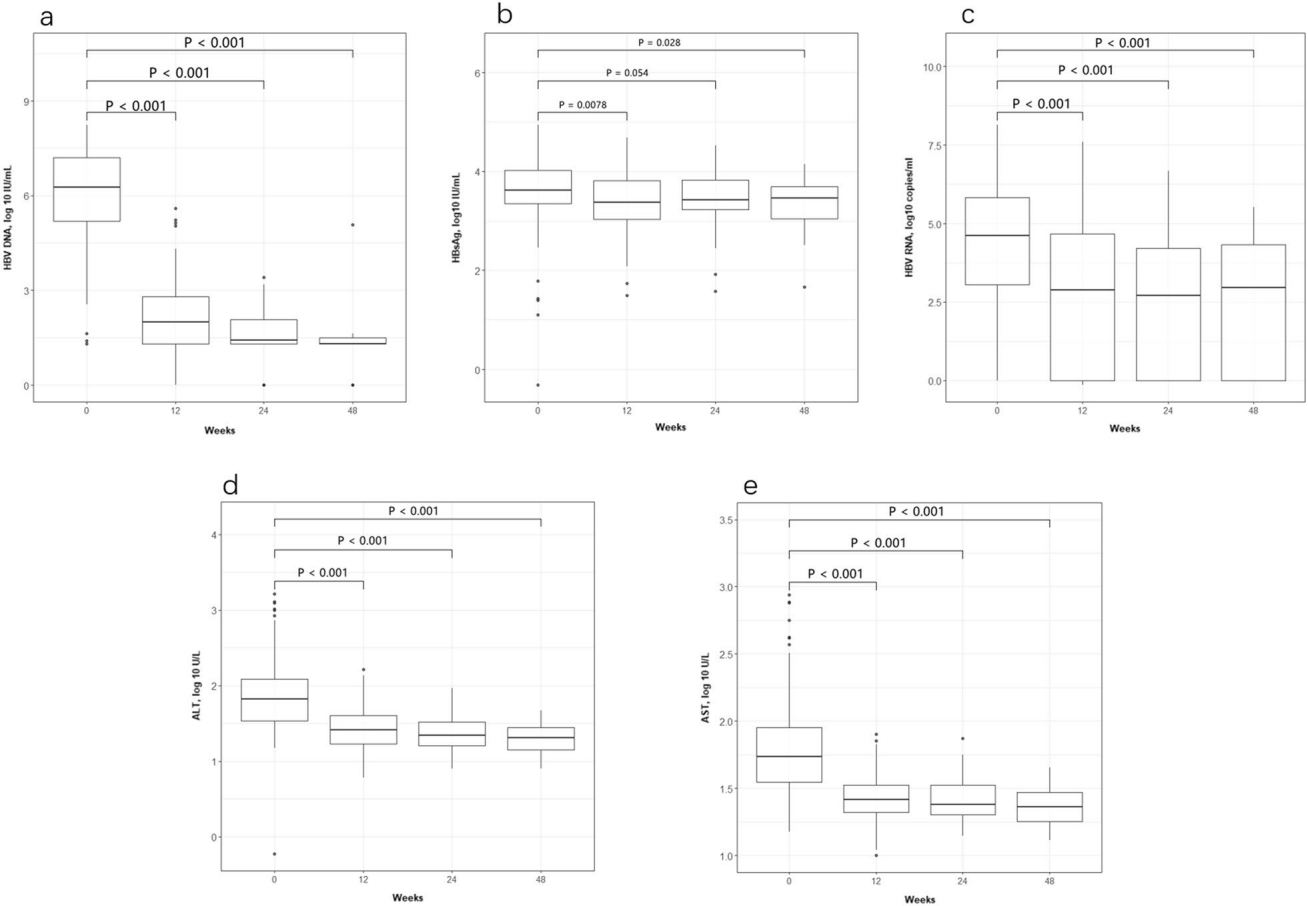
Diagrammatic presentation of changes in individual serological and virological markers in different follow-up time point. **a** HBV RNA viral load [log10 copies/ml]. **b** HBV DNA viral load [log10 IU/ml]. **c** HBsAg levels [log10 IU/mL]. **d** ALT levels [log10 IU/L]. **e** AST levels [log10 IU/L]

The total HBV serological markers and biochemical indicators presented a decreasing trend from baseline to 48 weeks. As shown in Fig. 3a, the mean level of serum HBV RNA was 4.62 (IQR: 3.05–5.82) log_10_ copies/mL at baseline; and the mean levels of serum HBV RNA at weeks 12, 24, and 48 were 2.88 (IQR: 0–4.67), 2.71 (IQR: 0–4.22) and 2.96 (IQR: 0–4.32) log_10_ copies/ mL, respectively (*P* < 0.05). The mean level of serum HBV DNA was 6.26 (IQR: 5.20–7.21) log_10_ IU/mL at baseline, and the mean levels of serum HBV DNA at weeks 12, 24, and 48 was 1.99 (IQR: 1.30–2.79), 1.41 (IQR: 1.30–2.07) and0.00 (IQR: 0.00–1.50) log_10_ IU/ mL (*P* < 0.05) (Fig. 3b). The mean level of serum HBsAg was 3.62 log_10_ IU/mL at baseline, and the mean level of serum HBsAg at weeks 12, 24, and 48 was 3.37, 3.43, and 3.46 log_10_ IU/ mL (Fig. 3c). Furthermore, during 48 weeks of first-line NAs therapy, biochemical indicators such as ALT and AST were gradually normal in Fig. 3d, e. In summary, the current long-time NA therapy can induce viral suppression and normalization of liver function.

### Correlation between HBV serological markers during 48 weeks of NA treatment therapy

The correlation coefficients between the viral biomarkers upon 12, 24, and 48 weeks of NA therapy were analyzed (Table 3). In general, HBV RNA showed a strong linear correlation with HBV DNA upon 12, 24, and 48 weeks of NA treatment (r = 0.640, 0.715, and 0.656, respectively, *P* < 0.05), and a weakened correlation between HBV RNA and HBsAg was still preserved at 12 and 24 weeks of NA treatment (r = 0.440 and 0.438, respectively, *P* < 0.05). However, at 48 weeks of NA treatment, there was no correlation between HBV RNA and HBsAg (r = 0.216, *P* = 0.252). In addition, the correlation between HBV DNA and HBsAg was still preserved upon 12 and 24 weeks (r = 0.519 and 0.398, respectively, *P* < 0.05), but not upon 48 weeks of NA treatment (r = 0.094, *P* = 0.622).

**Table 3:** Correlation between HBV serological markers in NA-treated patients

	HBV DNA	HBsAg
*Week 12 of NA therapy (n = 59)*		
HBV RNA	0.640(*P* < 0.05*)	0.440(*P* < 0.05*)
HBV DNA	–	0.519(*P* < 0.05*)
*Week 24 of NA therapy (n = 46)*		
HBV RNA	0.715(*P* < 0.05*)	0.438(*P* < 0.05*)
HBV DNA	–	0.398(*P* < 0.05*)
*Week 48 of NA therapy (n = 30)*		
HBV RNA	0.656(*P* < 0.05*)	0.216(*P* = 0.252)
HBV DNA	–	0.094(*P* = 0.622)

*P* < 0.05 is considered significant difference between two groups

### Subgroup analysis of correlation between HBV serological markers during 48 weeks of NA treatment therapy

When subgroup analysis is performed for HBeAg-positive and HBeAg-negative patients separately, the linear correlation between serum HBV RNA and HBV DNA & HBsAg was stronger in HBeAg-positive than in HBeAg-negative patients. The specific results are shown in Table 4. In HBeAg-positive patients, HBV RNA correlated with HBV DNA (at week 12: r = 0.594, week 24: r = 0.792, and week 48: r = 0.770; all *P* < 0.05) and with HBsAg (at week 12: r = 0.540, *P* < 0.05; week 24: r = 0.377, *P* < 0.05; and week 48: r = 0.201, *P* = 0.383), but not in HBeAg-negative patients. Furthermore, we also analyzed the correlation coefficients between the viral biomarkers in CHB and LC patients. In CHB patients, HBV RNA correlated with HBV DNA (at week 12: r = 0.686 week 24: r = 0.703, and week 48: r = 0.609; all *P* < 0.05) and with HBsAg (at week 12: r = 0.423 *P* < 0.05; week 24: r = 0.440, *P* < 0.05; and week 48: r = 0.031, *P* = 0.894). In LC patients, HBV RNA correlated with HBV DNA (at week 12: r = 0.600, *P* < 0.05; week 24: r = 0.736, *P* < 0.05 and week 48: r = 0.934, *P* < 0.05) and HBsAg (at week 12: r = 0.608, *P* < 0.05; week 24: r = 0.482, *P* = 0.226; and week 48: r = 0.627, *P* = 0.071) (Additional file 4: Table S1).

**Table 4:** Correlation between HBV serological markers in NA-treated patients according to HBeAg Status

	HBeAg-positive		HBeAg-negative	
	HBV DNA	HBsAg	HBV DNA	HBsAg
*Week 12 of NA therapy*				
HBV RNA	0.594(*P* < 0.05*)	0.540(*P* < 0.05*)	0.444(*P* < 0.05*)	0.358(*P* = 0.094)
HBV DNA	–	0.534(*P* < 0.05*)	–	0.551(*P* < 0.05*)
*Week 24 of NA therapy*				
HBV RNA	0.792(*P* < 0.05*)	0.377(*P* < 0.05*)	− 0.208(*P* = 0.517)	0.131(*P* = 0.685)
HBV DNA	–-	0.381(*P* < 0.05*)	–-	0.021(*P* = 0.949)
*Week 48 of NA therapy*				
HBV RNA	0.770(*P* < 0.05*)	0.201(*P* = 0.383)	0.230(*P* = 0.551)	0.297 (*P* = 0.438)
HBV DNA	–	0.011(*P* = 0.963)	–	0.365(*P* = 0.334)

*P* < 0.05 is considered significant difference between two groups

## Discussion

The management of chronic hepatitis B is an arduous task. HBV RNA has been regarded as a noninvasive surrogate tool for comprehensive antiviral therapy evaluation [15]. Significantly, patients with chronic hepatitis B virus infection need longitudinal serological and virological monitoring at different stages of the disease when receiving first-line oral NA antiviral treatment [16]. Our observational study's focus is to explore the longitudinal level of serum HBV RNA and its correlation with traditional serological markers within 48 weeks of NA antiviral treatment. The follow-up time points included baseline, 12 weeks, 24 weeks, and 48 weeks. This research showed details of HBV RNA under different stages of CHB natural history, from baseline to different treatment time points receiving NAs therapy.

In our study, at baseline, serum HBV RNA correlated well with HBV DNA and HBsAg (r = 0.602 and 0.502.; respectively. *P* < 0.05), which is consistent with our previous study [17]. Meanwhile, a moderate positive correlation between HBV RNA and HBV DNA (r = 0.445 and 0.478; respectively. *P* < 0.05) in HBeAg-positive and HBeAg-negative subgroups. However, in the HBeAg-negative subgroup, no significant correlation between HBV RNA or HBV DNA and HBsAg could be detected. This just confirmed the finding that serum HBsAg levels in HBeAg-negative are not associated with HBV replication activity markers, and there is a disparity between HBsAg and HBV replication in HBeAg-negative patients. This phenomena can be partly explained by the different HBsAg sources between HBeAg-positive and HBeAg-negative patients. In HBeAg-negative patients, HBsAg is thought to be mainly transcribed by integrated HBV genome fragments rather than cccDNA [18].

Notably, we also investigated the reverse transcriptional efficiency of pgRNA in the treatment-naïve chronic HBV infection cohort, as reflected by the ratio of serum HBV RNA to HBV DNA. Our research indicated that the reverse transcriptional efficiency was lower in HBeAg-positive patients than in HBeAg-negative patients (0.59 vs. 0.66; *P* < 0.001). This conclusion is consistent with the report of Hao Liao et al. [14], which means that with HBeAg seroconversion, the reverse transcriptional efficiency of RNA increased. In the same way, there was no significant difference in reverse transcriptional efficiency of RNA between the CHB and LC subgroup (0.59 vs. 0.62, *P* = 0.14).

Our study suggested that the proportions of patients with detectable HBV DNA, HBV RNA, and HBsAg during NA therapy in the cohort. To our knowledge, this is a detailed perspective report on the kinetics of HBV virological indicators within 48 weeks of NAs treatment. We found that the level of HBV DNA and HBV RNA decreased in varying degrees, and ALT/AST level tended to be normal. No case of HBsAg seroclearance was observed in our cohort. Early after NA therapy, HBV RNA would become higher than HBV DNA as showen in our cohort who were treated NAs for 12 weeks. This reason is that NAs can inhibit reverse transcription of pgRNA. While DNA synthesis is blocked, pgRNA will be accumulated transient and released into the circulation. Moreover, during a longer NA therapy, nuclear recycling of HBV DNA is impaired, leading to gradual decline in cccDNA pool, and thus serial reduction in pgRNA formation. Therefore, although serum HBV RNA would still be higher than serum HBV DNA during NA treatment, the level of HBV RNA will gradually decline with longer duration of NA therapy. Therefore, after 48 weeks of therapy, 37% of patients had detectable HBV DNA, whereas 67% had detectable HBV RNA. This finding is the same as a recently published study showing that all patients had achieved undetectable HBV DNA status, and 73% had detectable RNA [19]. These evidence supports that HBV RNA may serve as a better surrogate marker for cccDNA activity in virally suppressed patients receiving NAs therapy. Furthermore, at week 48 of antiviral therapy, median serum HBV RNA and HBsAg were still detectable at a median level of 2.96 (IQR: 0.00–4.32) log_10_ copies/mL and 3.46 (IQR:3.04–3.70) log_10_ IU/mL. This result is consistent with previous reports that the median level serum HBV RNA was 2.85 (IQR: 2.07–4.46) log_10_ copies/mL [20]. It also indirectly proves the reliability of HBV RNA in clinical detection compared with other HBV virological indicators.

Compared with baseline, at 12, 24, and 48 weeks of NA therapy, the median levels of serum HBV RNA were higher than HBV DNA by 1–2 log_10_. The explanation is that NAs treatment can effectively inhibit the HBV RNA reverse transcription and the follow-up degradation by RNase H activity of HBV polymerase (P protein). Then the RNA in core particles would still be enveloped and released into the serum, thereby increasing the level of HBV RNA virions. [21]. Therefore, NA therapy needs to last for a long time, nuclear recycling of HBV DNA via nucleocapsids into the host nucleus is impaired, leading to a gradual decline in the cccDNA pool and reduced the number of infected hepatocytes and thus a reduction in HBV RNA [22]. Despite the use of first-line NA, longer or lifetime treatment remains the norm; irregular drug withdrawal will lead to disease progression. Even when HBV DNA is effectively suppressed, we need alternative HBV serological markers such as HBV RNA to detect cccDNA transcriptional activity and gauge the therapy duration.

Our findings complement other recent reports of the profile and correlation of HBV RNA in serum. We observed the detailed dynamic changes and correlations of RNA within 48 weeks after NA treatment. Clinical patients are anxious in the initial phase of N.A. antiviral therapy, and our data can help patients more clearly understand the process of the disease, actively carry out standardized antiviral treatment, and improve their quality of life. In addition, the new antiviral agents upcoming, such as core protein allosteric modulators (CpAMs) and ribonucleic acid interfering (RNAi) gene silencers, are usually used in combination with NA therapy [23]. Our finding provides a basic understanding of the dynamic changes of HBV RNA under NA therapy within 48 weeks, which will play a supporting role in evaluating the efficacy of new agents' combined use in the future.

This study has three main limitations. First, because liver biopsies were not performed in our cohort, we could not directly analyze the correlation between intrahepatic cccDNA and HBV serological markers; moreover, due to the invasive and sampling errors of liver biopsy, there is no standardized cccDNA quantification method. Second, our follow-up cohort was relatively small, and the follow-up time for NA treatment was relatively short; a larger cohort and longer follow-up are necessary to evaluate HBV RNA more accurately. Third, our study is only a single-center study; a multi-center study should be conducted to confirm the results further.

## Conclusion

This research showed details of HBV RNA under different stages of chronic HBV infection natural history, from baseline to different treatment time points receiving NAs therapy, which will help us to assess HBV RNA clinical potentials. Our results indicate that serum HBV RNA decreased 1.66 log_10_ copies/mL from baseline to week 48 in chronic HBV infected patients receiving NAs therapy. In addition, Serum HBV RNA was positive in 67% of patients at 48 weeks of NAs therapy. Moreover, serum HBV RNA and HBsAg is significantly correlated at baseline, but this correlation disappeared during treatment, which suggests that serum HBV RNA could be a new marker for chronic Hepatitis B virus infection especially in patients whose serum HBV DNA cannot be detected receiving NAs therapy.

## Supplementary Information

**Additional file 1: Fig. S1:** The correlation plots for HBV serological markers in treatment-naïve patients. (a) HBV RNA and HBV DNA; (b) HBV RNA and HBsAg; (c) HBV DNA and HBsAg.

**Additional file 2: Fig. S2:** The correlation plots for HBV serological markers in treatment-naïve patients according to HBeAg status. (a) HBV RNA and HBV DNA in HBeAg-postive subgroup; (b) HBV RNA and HBsAg in HBeAg-postive subgroup; (c) HBV DNA and HBsAg in HBeAg-postive subgroup; (d) HBV RNA and HBV DNA in HBeAg-negative subgroup; (e) HBV RNA and HBsAg in HBeAg-negative subgroup; (f) HBV DNA and HBsAg in HBeAg-negative subgroup.

**Additional file 3: Fig. S3:** The correlation plots for HBV serological markers in treatment-naïve patients according to clinical diagnose. (a) HBV RNA and HBV DNA in CHB subgroup; (b) HBV RNA and HBsAg in CHB subgroup; (c) HBV DNA and HBsAg in CHB subgroup; (d) HBV RNA and HBV DNA in LC subgroup; (e) HBV RNA and HBsAg in LC subgroup; (f) HBV DNA and HBsAg in LC subgroup.

**Additional file 4: Table S1:** Correlation between HBV serological markers in NA-treated patients according to clinical diagnosis.

## Data Availability

The datasets used in the study are available from the corresponding author upon reasonable request. All authors read and approved the final manuscript.

## Abbreviations

ALT: Alanine amino transferase
AST: Aspartate amino transferase
cccDNA: Covalently closed circular DNA
CHB: Chronic hepatitis B
HBeAg: Hepatitis B e antigen
HBsAg: HBV surface antigen
HBV: Hepatitis B virus
pgRNA: Pregenomic RNA
LSM: Liver stiffness measurement
