# Supplementary figures and images for: Levels of HBV RNA in chronic HBV infected patients during first-line nucleos(t)ide analogues therapy
Source: Infect Agent Cancer. 2022 Dec 7;17:61. doi: 10.1186/s13027-022-00473-9 (PMC9727898; doi:10.1186/s13027-022-00473-9)

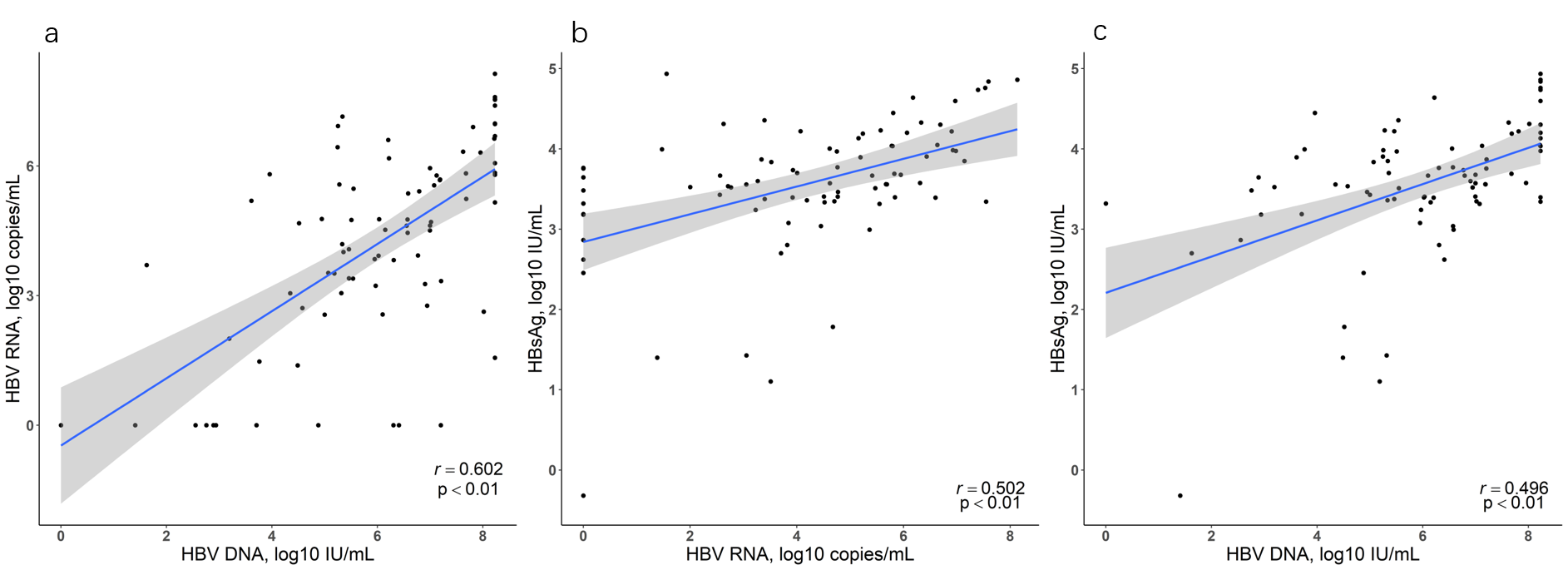

Supplement: Supplementary file 1 — Additional file 1: Fig. S1: The correlation plots for HBV serological markers in treatment-naïve patients. (a) HBV RNA and HBV DNA; (b) HBV RNA and HBsAg; (c) HBV DNA and HBsAg. [file 13027_2022_473_MOESM1_ESM.png]

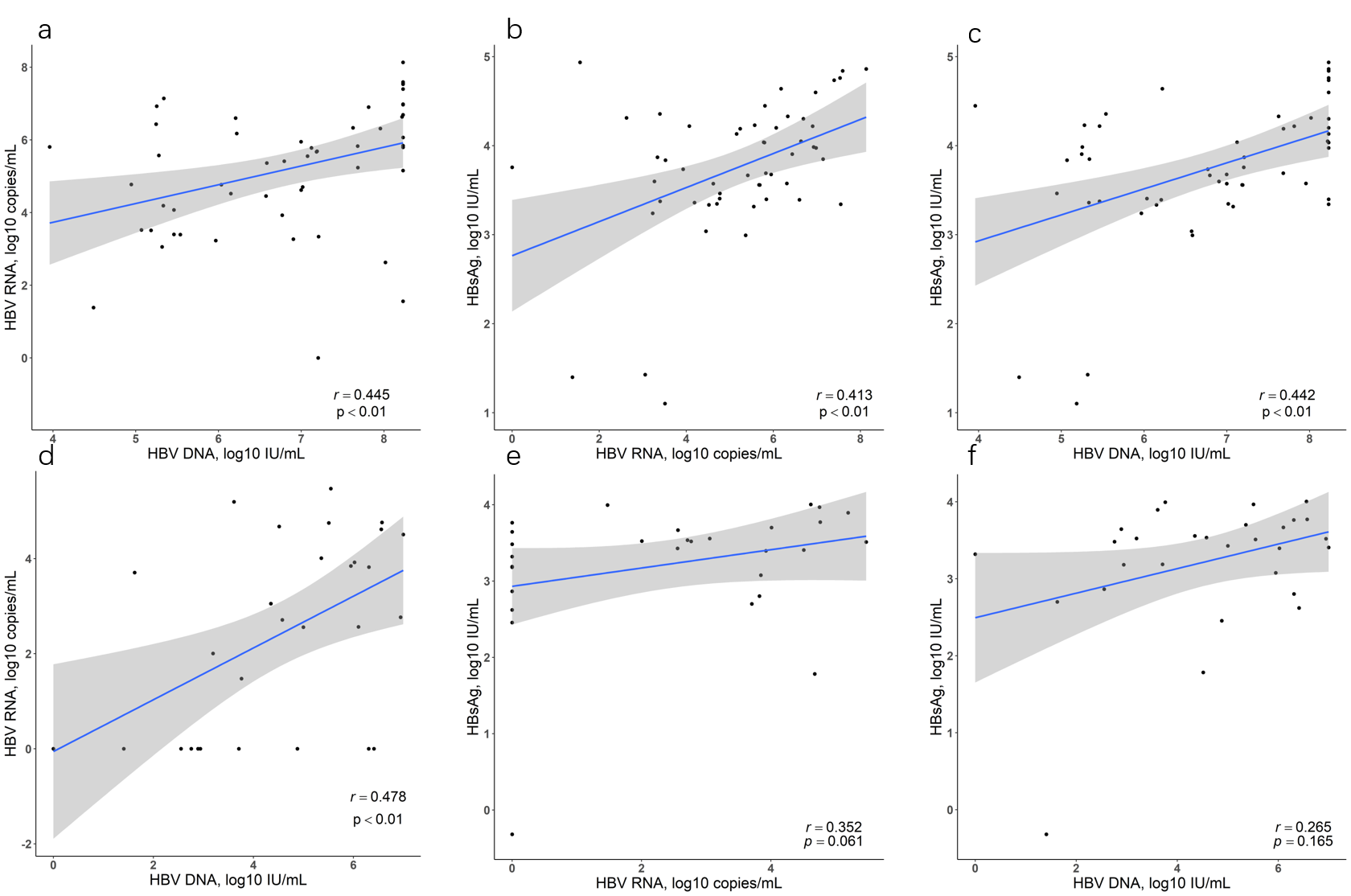

Supplement: Supplementary file 2 — Additional file 2: Fig. S2: The correlation plots for HBV serological markers in treatment-naïve patients according to HBeAg status. (a) HBV RNA and HBV DNA in HBeAg-postive subgroup; (b) HBV RNA and HBsAg in HBeAg-postive subgroup; (c) HBV DNA and HBsAg in HBeAg-postive subgroup; (d) HBV RNA and HBV DNA in HBeAg-negative subgroup; (e) HBV RNA and HBsAg in HBeAg-negative subgroup; (f) HBV DNA and HBsAg in HBeAg-negative subgroup. [file 13027_2022_473_MOESM2_ESM.png]

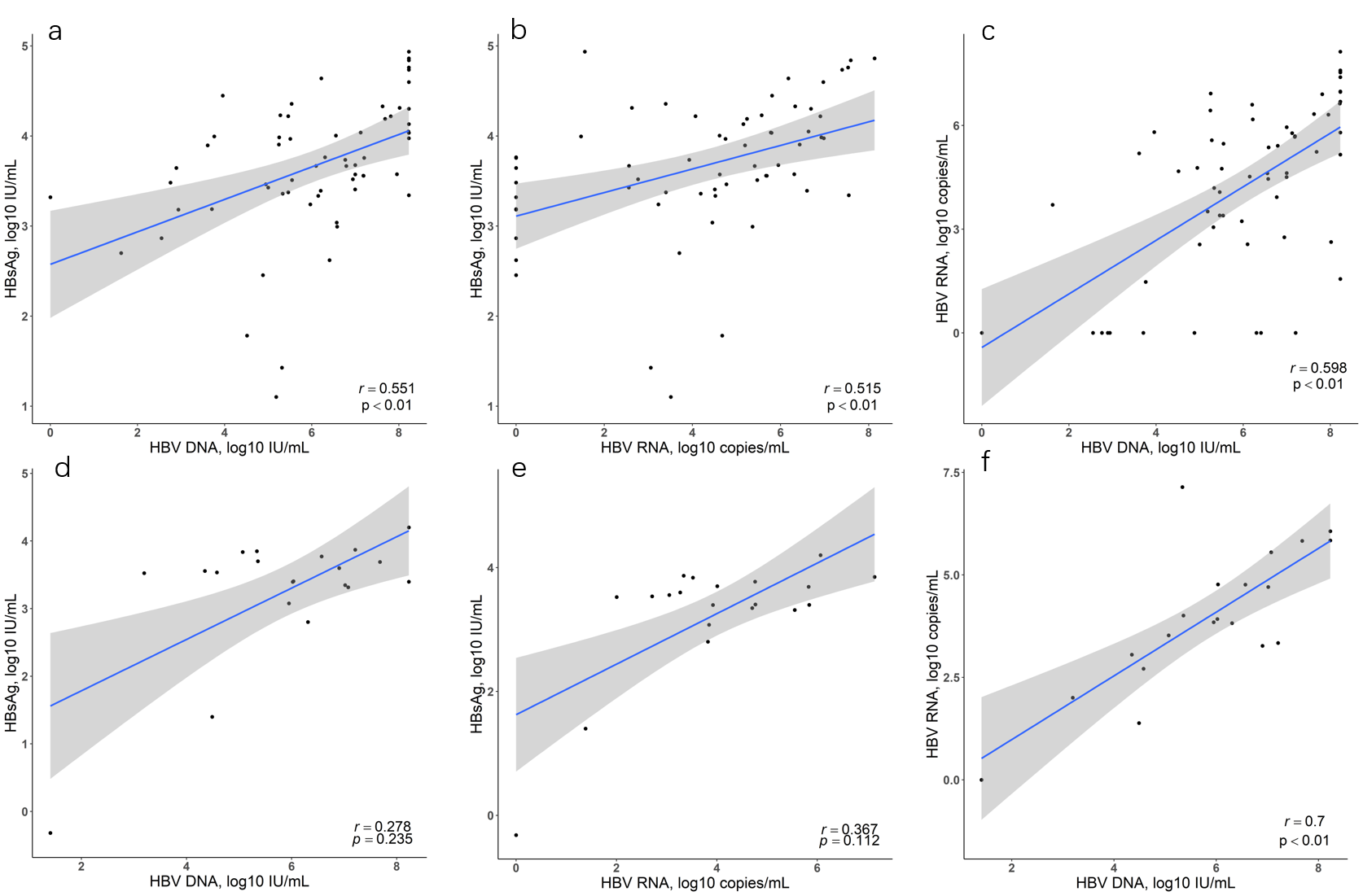

Supplement: Supplementary file 3 — Additional file 3: Fig. S3: The correlation plots for HBV serological markers in treatment-naïve patients according to clinical diagnose. (a) HBV RNA and HBV DNA in CHB subgroup; (b) HBV RNA and HBsAg in CHB subgroup; (c) HBV DNA and HBsAg in CHB subgroup; (d) HBV RNA and HBV DNA in LC subgroup; (e) HBV RNA and HBsAg in LC subgroup; (f) HBV DNA and HBsAg in LC subgroup. [file 13027_2022_473_MOESM3_ESM.png]
