# Supplementary material for: Levels of HBV RNA in chronic HBV infected patients during first-line nucleos(t)ide analogues therapy
Source: Infect Agent Cancer. 2022 Dec 7;17:61. doi: 10.1186/s13027-022-00473-9 (PMC9727898; doi:10.1186/s13027-022-00473-9)
Supplement: Supplementary file 4 — Additional file 4: Table S1: Correlation between HBV serological markers in NA-treated patients according to clinical diagnosis. [file 13027_2022_473_MOESM4_ESM.docx]

Table S1 Correlation between HBV serological markers in NA-treated patients according to clinical diagnosis

|  | CHB | | LC | |
| --- | --- | --- | --- | --- |
|  | HBV DNA | qHBsAg | HBV DNA | qHBsAg |
| Week 12 of NA therapy |  |  |  |  |
| HBV RNA | 0.686(*P*<0.05*) | 0.423(*P*<0.05*) | 0.600(*P*<0.05*) | 0.608(*P*<0.05*) |
| HBV DNA | --- | 0.538(*P*<0.05*) | --- | 0.442(*P*=0.130) |
| Week 24 of NA therapy |  |  |  |  |
| HBV RNA | 0.703 (*P*<0.05*) | 0.440(*P*<0.05*) | 0.736(*P*<0.05*) | 0.482(*P*=0.226) |
| HBV DNA | --- | 0.443 (*P*<0.05*) | --- | 0.051(*P*=0.905) |
| Week 48 of NA therapy |  |  |  |  |
| HBV RNA | 0.609(*P*<0.05*) | 0.031(*P*=0.894) | 0.934(*P*<0.05*) | 0.627 (*P*=0.071) |
| HBV DNA | --- | 0.012(*P*=0.958) | --- | 0.456(*P*=0.217) |

*P*<0.05 is considered significant difference between two groups.
